# Tetraspanin CD9 Regulates Cell Contraction and Actin Arrangement via RhoA in Human Vascular Smooth Muscle Cells

**DOI:** 10.1371/journal.pone.0106999

**Published:** 2014-09-03

**Authors:** Michael J. Herr, Scott E. Mabry, Lisa K. Jennings

**Affiliations:** 1 The Vascular Biology Center of Excellence and the Department of Internal Medicine, University of Tennessee Health Science Center, Memphis, Tennessee, United States of America; 2 Department of Microbiology, Immunology, and Biochemistry, University of Tennessee Health Science Center, Memphis, Tennessee, United States of America; 3 Department of Surgery, University of Tennessee Health Science Center, Memphis, Tennessee, United States of America; 4 Joint Program of Biomedical Engineering, University of Tennessee Health Science Center and the University of Memphis, Memphis, Tennessee, United States of America; 5 CirQuest Labs, LLC, Memphis, Tennessee, United States of America; King's College London, United Kingdom

## Abstract

The most prevalent cardiovascular diseases arise from alterations in vascular smooth muscle cell (VSMC) morphology and function. Tetraspanin CD9 has been previously implicated in regulating vascular pathologies; however, insight into how CD9 may regulate adverse VSMC phenotypes has not been provided. We utilized a human model of aortic smooth muscle cells to understand the consequences of CD9 deficiency on VSMC phenotypes. Upon knocking down CD9, the cells developed an abnormally small and rounded morphology. We determined that this morphological change was due to a lack of typical parallel actin arrangement. We also found similar total RhoA but decreased GTP-bound (active) RhoA levels in CD9 deficient cells. As a result, cells lacking a full complement of CD9 were less contractile than their control treated counterparts. Upon restoration of RhoA activity in the CD9 deficient cells, the phenotype was reversed and cell contraction was restored. Conversely, inhibition of RhoA activity in the control cells mimicked the CD9-deficient cell phenotype. Thus, alteration in CD9 expression was sufficient to profoundly disrupt cellular actin arrangement and endogenous cell contraction by interfering with RhoA signaling. This study provides insight into how CD9 may regulate previously described vascular smooth muscle cell pathophysiology.

## Introduction

Smooth muscle cells (SMC) localized in the medial layer of the arterial wall are primarily responsible for regulating the physiomechanical properties of arteries. These cells are not terminally differentiated and retain the ability to transform their phenotype from contractile or differentiated to synthetic or dedifferentiated. The switch from a contractile to synthetic phenotype is a well-studied though complex occurrence primarily characterized by a change in cell morphology from elongated to more rounded cells and by a decrease in the expression of two or more smooth muscle cell marker proteins [1], [2]. Vascular smooth muscle cells (VSMC) in the synthetic state are associated with coronary artery diseases including atherosclerosis and restenosis as well as with hypertension.

Understanding the mechanisms that control VSMC phenotype switching during vascular development and in vascular disease is an intense area of investigation. The importance of cell surface proteins, specifically integrins and tetraspanins, and their regulation of interactions with the extracellular matrix (EMC) have been previously demonstrated to play a relevant role in vascular cell biology [3]–[7]. Tetraspanins are ubiquitously expressed in vascular and hematopoietic cells and have implications in multiple physiologic and pathologic functions, yet they are understudied in the field of vascular biology [7].Tetraspanins function primarily as cell surface organizers and play an integral role in the potentiation of cellular responses from the extracellular environment in multiple cell types. Importantly, it has been demonstrated that the action of integrins, fundamental cell-cell and cell-ECM interacting proteins, is dependent on their interaction with tetraspanins [8].

One prominent member of the tetraspanin family, CD9, has been implicated in multiple essential cellular processes including proliferation [9], migration [10], and neointimal formation [6]. Specifically, we and others have demonstrated an elevated expression level of tetraspanin CD9 on the cell surface of cultured VSMCs in the synthetic state [6], [11]. The expression of CD9 directly correlated with the dedifferentiated phenotype of smooth muscle cells. Blockade or stimulation of CD9 using monoclonal antibodies resulted in the reduction or propagation of these phenotypes, respectively. However, there has not been an explanation as to how CD9 regulates the mechanical and phenotypic properties of these cells [12], [13].

The present study utilized a human model of arterial function, human aortic smooth muscle cells (HAOSMC), to specifically investigate the importance CD9 expression in regulating VSMC phenotypes. We found that CD9 knockdown resulted in pronounced morphologic changes and altered cellular actin arrangement. Furthermore, lack of CD9 reduced the highly coordinated endogenous contractile capabilities of HAOSMC. We identified GTP-bound RhoA (active RhoA) levels to be significantly diminished in cells lacking CD9. Restoration of RhoA activity in the CD9 deficient cells was sufficient to reestablish the contractile phenotype. Conversely, inhibition of active RhoA resulted in a contractile phenotype that mimicked CD9 deficient cells. The results reported here outline a previously unexplained phenomenon by which CD9 has a key role in regulating endogenous VSMC contraction via RhoA activation.

## Materials and Methods

### Reagents and Antibodies

Smooth muscle cell basal media (SmBM), fetal bovine serum (FBS), recombinant human epidermal growth factor (rhEGF), recombinant human fibroblast growth factor (rhFGF), recombinant human insulin, and gentamicin sulfate/amphotericin-B were purchased from Lonza (CC-3182, Walkersville, MD). Antibodies to anti-human CD9 (mAb7) were generated in our laboratory as previously described [14]. Anti-human CD81 was purchased from Santa Cruz Biotechnology (sc-7637, Santa Cruz, CA), and anti-human CD151 was purchased from BD Biosciences (556056, Durham, NC). Polybrene (H9268), puromycin (P8833), and anti-human β-tubulin (T2200), IgG (M9269), and FITC-conjugated anti-mouse (F2012) antibodies were purchased from Sigma Aldrich (St. Louis, MO). The horseradish peroxidase (HRP)-conjugated secondary antibodies anti-rabbit (NA934VS) and anti-mouse (NA931VS) were purchased from GE Healthcare (Pittsburgh, PA). Alexa-fluor 594 conjugated phalloidin (A12381) and Lipofectamine 2000 (11668) was purchased from Life Technologies (Grand Island, NY). The RhoA activator (CN03) and the RhoA inhibitor (CT04) were purchased from Cytoskeleton (Denver, CO).

### Generation of Lentiviral shRNA

CD9 lentiviral shRNA was generated in the viral vector core laboratory at the University of Tennessee Health Science Center (UTHSC, Memphis, TN). Five separate pLKO.1 CD9 shRNA lentiviral vector plasmids were obtained from Open Biosystems (Thermo Scientific, Pittsburgh, PA) and the plasmids were propagated in DH5α E. Coli in LB+Carbencillin containing media. Plasmid efficacy was determined by transient transfection using Lipofectamine 2000 (Invitrogen, Grand Island, NY) per the manufacturer's protocol. Western blot was used to determine the amount of CD9 knockdown as described below (data not shown). One CD9 shRNA plasmid was more effective than the other four and was used to generate lentiviral shRNA. pLKO.1 CD9 shRNA or scrambled control (Ctr) shRNA was co-transfected into 293FT cells using ViraPower mix kit (Invitrogen, Grand Island, NY). Cell culture supernatants were harvested 60 h after transfection, filtered through 0.45 µM filters, and the lentivirus was concentrated by ultracentrifugation at 50,000 x g for 3 h.

### Cell Culture

Primary human aortic smooth muscle cells (HAOSMC) were purchased from Lonza at passage 3. HAOSMC were cultured in a humidified incubator at 37°C and 5% CO_2_ in SmBM containing a final concentration of 5% FBS, 0.5 ng/ml rhEGF, 2 ng/ml rhFGF, 5 µg/ml insulin, and 50 µg/ml gentamicin sulfate/amphotericin-b (serum-supplemented SmBM). Cells were maintained in the synthetic state by changing media every 48 h and passaging every 96 h. At passage 5, HAOSMC at 70% confluence were transduced in 6-well culture dishes using 1 ml of serum-free SmBM containing 10 µl of lentiviral shRNA and a final concentration of 10 µg/ml polybrene. Transduction was complete after 24 h and selection of cells positive for shRNA constructs was performed 48 h after transduction using a final concentration of 1.0 µg/ml puromycin. All successive experiments were conducted using Ctr or CD9 shRNA HAOSMC between passages 6 and 8.

### RNA Extraction and qRT PCR Analysis

RNA was extracted from HAOSMC using the RNeasy minikit (74104, Qiagen, Valencia, CA) per the manufacturer's instructions. The RNA quantity was estimated using a nanodrop spectrophotometer (Thermo Scientific, Rockford, IL) and RNA integrity was assessed using Agilent bioanalyzer 2100 (Santa Clara, CA). cDNA was made from 500ng of total RNA with an RNA integrity number greater than 9 using a cDNA synthesis kit from Applied Biosystems (4374966, Foster City, CA). The resulting cDNA was tested in triplicate by qRT-PCR using TaqMan chemistry (Roche, Indianapolis, IN) and a Lightcycler 480 system at the UTHSC Molecular Resource Center. Forward and reverse primers were designed and ordered from Sigma Aldrich, and primer efficiency was tested on a serial dilution of universal human RNA. Primers with an efficiency of greater than 1.90 were used in subsequent experiments. Human primer sequences were as follows: CD9 forward GAG GCA CCA AGT GCA TCA A; CD9 reverse AGC CAT AGT CCA ATG GCA AG; smooth muscle α-actin forward CTG TTC CAG CCA TCC TTC AT; smooth muscle α-actin reverse TCA TGA TGC TGT TGT AGG TGG T; SM22α forward CAG TGT GGC CCT GAT GTG; SM22á reverse CAC CAG CTT GCT CAG AAT CA; calponin forward CCA ACC ATA CAC AGG TGC AG; calponin reverse TCA CCT TGT TTC CTT TCG TCT T; RhoA forward GGG AGC TAG CCA AGA TGA AG; RhoA reverse GTA CCC AAA AGC GCC AAT C. Fold changes in mRNA expression were calculated from the resulting CT values from three independent experiments as described earlier [15].

### Western Blotting

Lysates of HAOSMC were made in a TX-100 lysis buffer (1% TX-100, 20mM Tris-HCl, 150mM NaCl, 0.5% deoxycholate, and 0.1% sodium dodecyl sulfate) including protease and phosphatase inhibitor cocktails purchased from Sigma Aldrich. Lysate concentration was determined using a colorimetric Bradford Assay and a standard curve. Equal concentrations of lysate mixed with 1/3^rd^ reducing or non-reducing buffer were loaded onto a SDS-polyacrylamide gel. The proteins were transferred to a polyvinyl difluoride membrane and non-specific sites of binding were blocked using 5% BSA in Tris-buffered saline with 1% Tween-20 (TBST) solution. Anti-human CD9 (mAb7, 1∶500) or anti-human β-tubulin (1∶20,000) was diluted in 5% BSA-TBST and incubated overnight at 4°C. After washing off the primary antibody with TBST, a HRP-conjugated anti-mouse antibody (1∶5,000) for mAb7 or anti-rabbit antibody (1∶50,000) for β-tubulin was diluted in 5% BSA-TBST, added to the blots, and incubated for 1 h at room temperature. The blots were again washed as described earlier and incubated with Pierce ECL 2 Western blotting substrate (80196, Thermo Scientific, Rockford, IL) for 5 min. X-ray film was used to detect chemiluminesence and the films were developed using a Konica Minolta X-ray machine. Band density was determined using NIH ImageJ software, and mAb7 band densities were normalized the corresponding to β-tubulin band densities and averaged.

### Flow Cytometry

HAOSMC were harvested and suspended at 5.0×10^5^ cells/ml in 5% goat serum-SmBM (blocking media), placed on ice, and incubated for 45 min to block non-specific binding. Primary antibodies were added at a final concentration of 5 µg/ml and allowed to incubate for 1 h. The cells were then washed three times by centrifugation at 3,000 RPM for 5 min, aspiration of the media, then suspending the resulting pellet in ice-cold phosphate-buffered saline (PBS, pH 7.4). The FITC-conjugated secondary antibody (1∶100) was diluted in blocking media, then added and allowed to incubate for 1 h. The cells were again washed three times and the pellet was suspended after the last wash in room temperature PBS, added to a test tube and the data was acquired using a FACS Calibur flow cytometer equipped with Cell Quest Pro software (Becton-Dickinson, Bedford, MA). The geometric mean fluorescence intensity values were averaged among three or more independent experiments.

### Measurement of the area of Crystal Violet Stained Cells

Control and CD9 shRNA HAOSMC were harvested, counted, and diluted to equal densities in serum-supplemented SmBM and 50,000 cells were added in 1 ml of media to a 12-well culture plate. After 2 hours of adhesion, the cells were washed twice with PBS and fixed and permeabilized in ice cold methanol. After 5 min, the methanol was washed off and a saturating amount of 0.05% crystal violet was added for 30 min. The crystal violet was aspirated and the cells were washed twice with distilled water and allowed to dry. Pictures were then taken at 10x and 40x magnifications using an inverted phase contrast microscope, a Sony 3CCD color video camera (Tokyo, Japan) and Scion Image software (Frederick, MD). The area of the cells at 40x magnification was measured in arbitrary units using NIH ImageJ software. This experiment was completed three independent times and a total of 25 cells from random fields of view from each experiment were measured to calculate the average area.

### Adhesion Assay

Control and CD9 shRNA cells were harvested, counted, and diluted to equal densities in serum-supplemented SmBM and 20,000 cells in 500 µl of media were added to each well of a 24-well cell culture plate. After 2 h, the media was aspirated and the cells were fixed and stained with crystal violet as described in the previous section. Cells were counted in 20 random fields of view at 10x magnification and the resulting numbers were averaged. Each adhesion assay experiment was repeated at least three independent times.

### Immunofluorescent staining of F-actin

Ctr and CD9 shRNA HAOSMC were harvested and seeded at low density onto glass coverslips in 6-well cell culture dishes in serum-supplemented SmBM. After 2 h of attachment in the presence or absence of any treatments, the media was aspirated and cells were fixed in 4% paraformaldehyde (PFA)-PBS solution. After 10 min incubation the 4% PFA-PBS solution was aspirated, and the cells were rinsed three times with PBS then permeabilized using 0.1% TX-100 in PBS for 5 min. The TX-100 solution was aspirated and the monolayer of cells was washed three times with 5% goat serum (GS) in PBS and incubated with 5% GS-PBS for 30 min to block any non-specific binding. Alexa-fluor-594 conjugated phalloidin (1∶50) in 5% GS-PBS was added to the coverslips and incubated for 30 min at room temperature. After aspirating and washing the monolayer of cells with PBS, the coverslips were inverted and mounted to glass slides using Vectashield hard set mounting medium (H-1400, Vector Laboratories, Burlingame, CA). Images were acquired using an Axioplan 2 fluorescent imaging microscope equipped with an AxioCam HRm camera (Zeiss, Oberkochen, Germany). Actin length was quantified using AxioVision software by measuring the length in microns of 10 random, well-defined filaments per cell for 5 cells for three independent experiments. The lengths were averaged and the results were presented as the mean ± standard deviation.

### Detection of Total and GTP-bound RhoA

Active and GTP-bound RhoA were measured from cell lysates using a total RhoA ELISA (BK150) or RhoA activation G-LISA kit (BK124) purchased from Cytoskeleton, Inc. (Denver, CO) per the manufacturer's instruction. Briefly, equal numbers of Ctr and CD9 shRNA cells were lysed at the indicated time points using the provided cell lysis buffer and protease inhibitor cocktail. Lysates were quantified using the precision red protein assay reagent by measuring the absorbance at 600nm on the Versamax plate reader. Lysates were diluted to 0.5mg/ml using lysis buffer and then loaded on to their respective ELISA or G-LISA plate for protein analysis. Results were obtained as absorbance values at 490nm and were converted to total protein levels using a standard curve obtained with each test.

### Collagen Gel Contraction Assay

Collagen gel contraction assays were purchased from Cell Biolabs (CBA-201, San Diego, CA) and used per the manufacturer's instructions. HAOSMC were harvested and the pellet was suspended in serum-free SmBM at 2.0×10^6^ cells/ml. For each assay, 100 µl of the cell suspension was mixed with 400 µl of neutralized collagen solution and added to one well of a 24-well cell culture plate and allowed to solidify for one h at 37°C. After polymerization, 1ml of complete media was added to the top of each collagen gel. After 48 h incubation, the gels were released by running a sterile pipette tip along the sides of the well. Some populations of cells were treated with either CN03 or CT04 2 h prior to release. The culture dish was then scanned immediately after being released (time 0, initial time point) and for 30 min intervals for 6 h. ImageJ software was used to measure the area of each collagen gel and the arbitrary units (AU) for each time point were used to calculate the exact percent contraction values from the initial time point using the following formula: [(AU at Initial Time Point – AU at Current Time Point)/AU at Initial Time Point]*100%.

### Statistical Analysis

Each experiment was performed at least three independent times. Samples were tested in duplicate or triplicate. The Student t-test or Mann Whitney U test was performed to compare normally distributed or non-normally distributed samples, respectively. For the contraction assays, a repeated measures t-test was used to compare the changes in contraction among two groups at each time point. Bars or points represent the mean result and error bars represent the standard deviation of the mean. Each test was run at a 95% confidence level and *P*-values less than 0.05 were considered statistically significant.

## Results

### CD9 deficiency results in altered cell morphology and SMC marker expression

**Figure 1:**
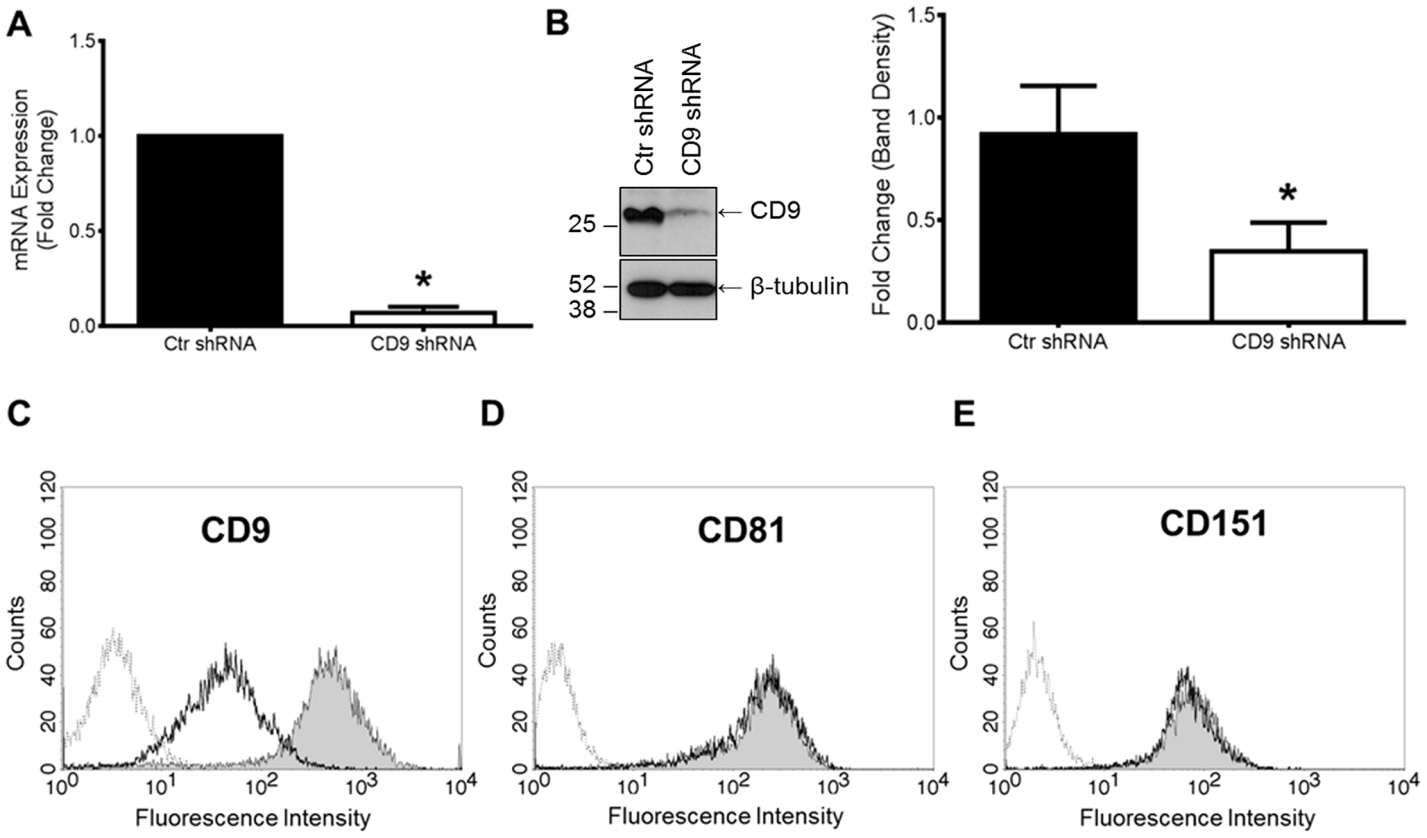
Knockdown of tetraspanin CD9 in human aortic smooth muscle cells (HAOSMCs) does not affect CD81 or CD151 cell-surface expression. **A,** The fold change in CD9 mRNA in control (Ctr) shRNA and CD9 shRNA transduced cells was measured using qRT-PCR (n = 5; *P<0.05). **B,** Whole cell expression of CD9 was measured by Western blotting using an anti-human CD9 monoclonal antibody (mAb7). A representative blot and quantification of three independent blots is presented (*P<0.05). C-E, Cell surface expression of CD9, CD81, and CD151 was measured on non-permeabilized HAOSMCs using flow cytometry. Dotted gray lines represent negative control IgG binding, shaded histograms represent Ctr shRNA HAOSMC, and open histograms represent CD9 shRNA binding of indicated anti-human tetraspanin antibodies. A representative histogram from three independent experiments is shown for each anti-human tetraspanin antibody.

Smooth muscle cells in culture have augmented CD9 expression that is directly correlated to their synthetic state [6], [11]. We used CD9 lentiviral shRNA to knockdown CD9 expression in human aortic smooth muscle cells (HAOSMC). Decreased CD9 expression in the CD9 shRNA HAOSMC was observed at the mRNA and protein levels when compared to cells treated with scrambled control (Ctr) lentiviral shRNA, (Fig. 1A and 1B). More importantly, flow cytometric analysis of non-permeabilized cells revealed that CD9 expression was reduced at the cell surface where it primarily functions (Fig. 1C). The expression of a closely related tetraspanin, CD81, and another highly functional tetraspanin, CD151, was not affected by CD9 knockdown at the mRNA or cell surface levels (Fig. 1D and 1E).

**Figure 2:**
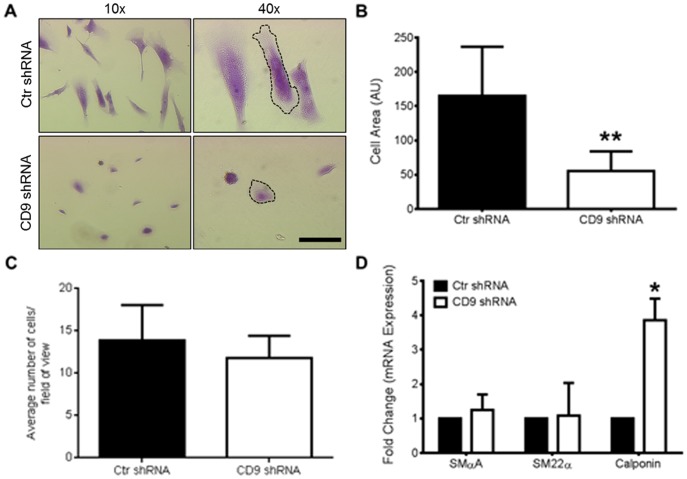
CD9 deficient HAOSMCs have an altered cell morphology, decreased cell size, and an increased level of calponin mRNA. **A,** Knockdown of CD9 results in profoundly smaller and more rounded cells as demonstrated by representative phase contrast images of crystal violet stained cells at different magnifications (n = 4 independent repeats; scale bar, 100 µm). **B,** Quantification of cell area of Ctr and CD9 shRNA HAOSMCs (n = 25 cells; **P<0.01). **C,** Ctr and CD9 shRNA HAOSMC adhesion at 2 h in culture as measured by counting cells and averaging cells per random field of view (NS = not significant). **D,** mRNA levels of SM α-actin, SM22α, and calponin were measured using qRT-PCR (n = 3; *P<0.05).

Knockdown of CD9 had a noticeably profound difference in cell morphology at all stages of observation of the cells in serum-supplemented culture media. This morphologic difference is demonstrated in figure Fig. 2A, by crystal violet staining the cells after plating in serum-supplemented media for 2 h. HAOSMC lacking CD9 were notably smaller as determined by cell area and more rounded than their Ctr shRNA treated counterparts (*P*<0.01; Fig. 2B). Reducing CD9 expression resulted in a cell morphology that resembled the dedifferentiated phenotype of SMCs [2].

This phenotype led us to explore whether or not the adhesive properties of these cells differed at the time the morphology was noticeably altered. We found no differences in adhesion of the Ctr and CD9 shRNA HAOSMC at the same time point (Fig. 2C, P = 0.0642). To further elucidate the phenotype of CD9 shRNA HAOSMCs, the expression levels of three smooth muscle cell marker proteins were measured. The mRNA expression of smooth muscle α-actin (SM α-actin) and SM22α were not significantly different between the two groups of cells (Fig. 2D). However, the expression of a third smooth muscle cell marker, calponin, was increased 4-fold (*P*<0.05; Fig. 2D).

### CD9 deficient HAOSMC have altered actin arrangement and decreased contractile capabilities due to RhoA mediated pathways

**Figure 3:**
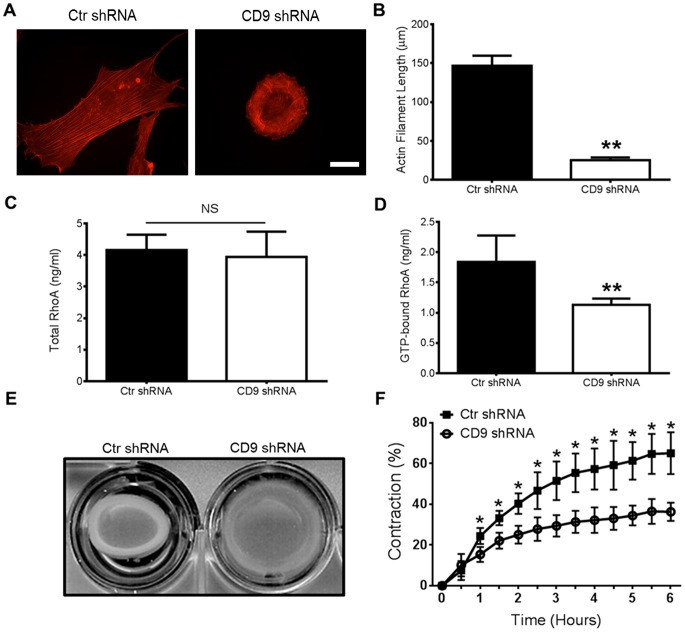
HAOSMCs lacking CD9 display altered actin arrangement and a decrease in active RhoA and collagen gel contraction. **A,** Ctr and CD9 shRNA cells were fixed, permeabilized and stained with Alexa-fluor 594 conjugated phalloidin and viewed using a immunofluorescent deconvolution microscope (scale bar, 50 µm). **B,** Quantification of actin filament length was performed using ImageJ analysis software (n = 15 cells; **P<0.01). **C** and **D,** Total and GTP-bound (active) RhoA was measured in whole cell lysates from Ctr and CD9 shRNA HAOSMC using an ELISA and the absorbance values were converted to concentration values from a standard curve (n = 3; NS =  not significant; **P<0.01). **E** and **F,** A representative image of a collagen gel contraction assay taken at 6 h and quantification of the percent contraction over time (n = 3/time point; *P<0.05 versus CD9 shRNA at indicated time point).

We hypothesized that decreased CD9 expression and the significant phenotypic transformation may be a consequence of changes in the cellular cytoskeleton. Alexa fluor-conjugated phalloidin and immunofluorescent microscopy were used to characterize changes in the stress fiber arrangement between Ctr and CD9 shRNA treated HAOSMC (Fig. 3A). The CD9 shRNA HAOSMC had a more circular arrangement of significantly shorter actin stress fibers whereas the Ctr shRNA HAOSMC had a more elongated and parallel arrangement of stress fibers (*P*<0.01; Fig. 3B). These findings suggested that CD9 may elicit the marked morphologic difference between the two cell types by regulating the stress fiber arrangement.

As it is well established that actin stress fiber arrangement is a direct consequence of RhoA activity [16], [17], we measured the amount of total and GTP-bound (active) RhoA in Ctr and CD9 shRNA HAOSMC using an enzyme linked immunosorbent assays. Total RhoA was not significantly different between the two groups (Fig. 3C); however, there was significantly less active RhoA detected in the CD9 deficient cells (*P*<0.05, Fig. 3D). We performed a collagen gel contraction assay to determine if the observed decrease in RhoA activity in CD9 shRNA cells had any effect on HAOSMC contraction. After 1 h, the Ctr shRNA cells were significantly more contractile and remained so for the entire assay (*P*<0.05; Fig. 3E and 3F). At the conclusion of the assay, CD9 shRNA HAOSMC were 30% less contractile than their Ctr shRNA counterparts. Contraction in both groups began to plateau after 4 h and remained stable for the duration of the assay.

### Activation of RhoA restores the CD9 shRNA HAOSMC phenotype

**Figure 4:**
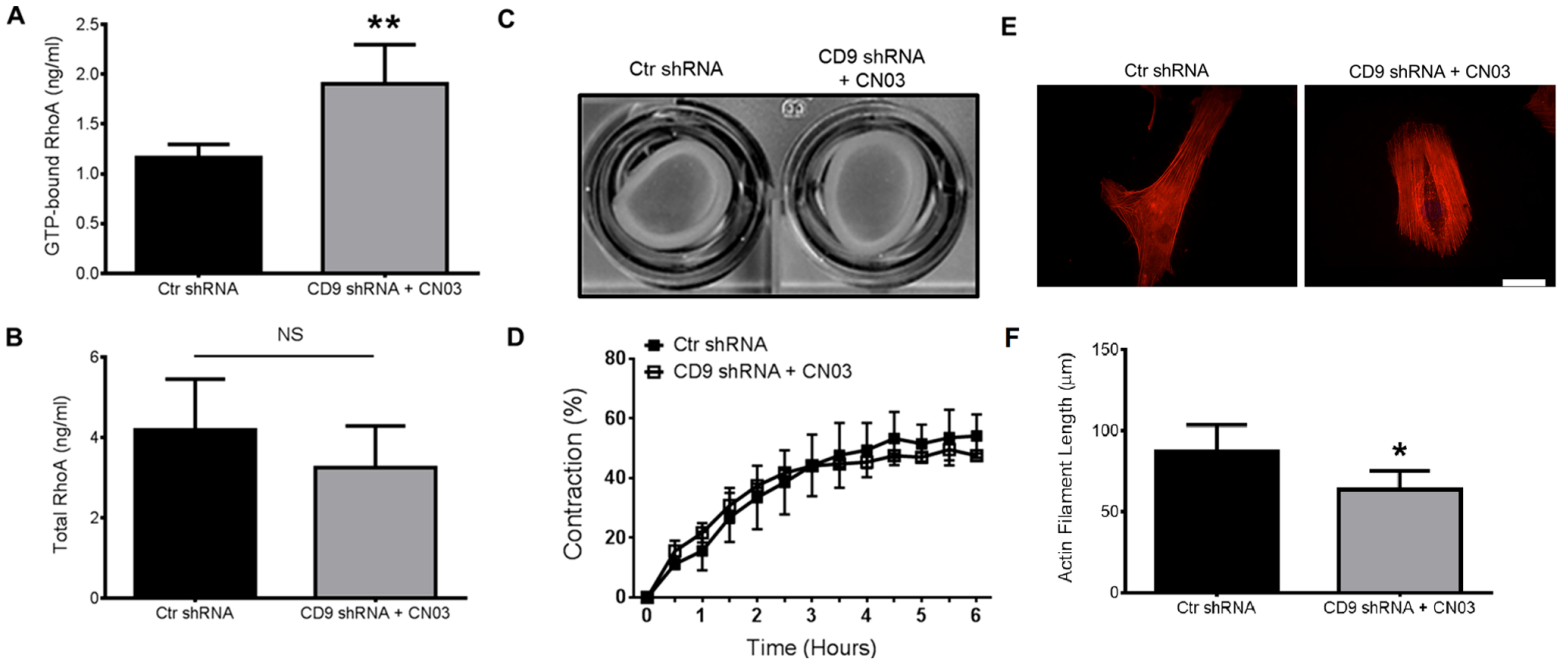
Activation of RhoA restores the contractile capabilities of CD9 shRNA HAOSMC and partially restores actin arrangement. **A** and **B,** GTP-bound (active) and total RhoA was quantitated using whole cell lysates of Ctr shRNA HAOSMC or CD9 shRNA HAOSMC treated with the RhoA activator CN03 (2.0 µg/ml) for two h (n = 3; NS = not significant; **P<0.01). **C** and **D,** CD9 shRNA cells were treated with CN03 (2.0 µg/ml) for two h prior to releasing the sides of a collagen gel contraction assay. A representative image of a collagen gel contraction taken at 6 h and quantification of collagen gel contraction from 0-6h is shown (n = 3/time point). **E,** A representative immunofluorescent stained image of f-actin arrangement from three independently repeated experiments in untreated Ctr shRNA and CD9 shRNA treated with 2.0 µg/ml of CN03 for two h (scale bar, 50 µm). **F,** Actin filament length was quantified using ImageJ analysis software (n = 15 cells; *P<0.05).

The observed deficiency of active RhoA and loss of the contractile phenotype in CD9 deficient HAOSMC led us to explore if there was a direct cause and effect between level of active RhoA and the measured contractile response. We utilized the commercially available, cell permeable RhoA activator CN03, which pairs the catalytic domain of bacterial cytotoxic necrotizing factor (CNF) toxin with a proprietary cell penetrating moiety. CN03 blocks intrinsic GTPase activity to sustain GTP-bound RhoA in treated cells [18]. Restoration of GTP-bound RhoA in CD9 shRNA HAOSMC by CN03 was confirmed (Fig. 4A) and did not significantly affect the levels of total RhoA (*P* = 0.19; Fig. 4B). Upon treatment with CN03, we observed an increase in the contractile capability of CD9 shRNA HAOSMC compared to untreated Ctr shRNA cells (Fig. 4C). The significant difference in contraction observed between the two cell groups was no longer obvious after treatment with CN03 (Fig. 4D). Comparison of the actin cytoskeleton of CN03 treated CD9 shRNA HAOSMC at 2 h revealed that actin stress fibers were arranged more parallel to one another than the untreated CD9 shRNA cells (Compare Figure 3A right panel with Fig. 4E left panel). However, the CN03 treated cells did not display the fully elongated stress fibers originally observed in the Ctr shRNA HAOSMC and were statistically significantly shorter (Fig. 4F; P = 0.0295). These data indicate that the absence of active RhoA upon knockdown of CD9 results in a lack of arranged actin, and that active RhoA is necessary for HAOSMC-mediated collagen gel contraction.

### Deficiency of Active RhoA or loss of total RhoA mimics the CD9 shRNA HAOSMC phenotype

**Figure 5:**
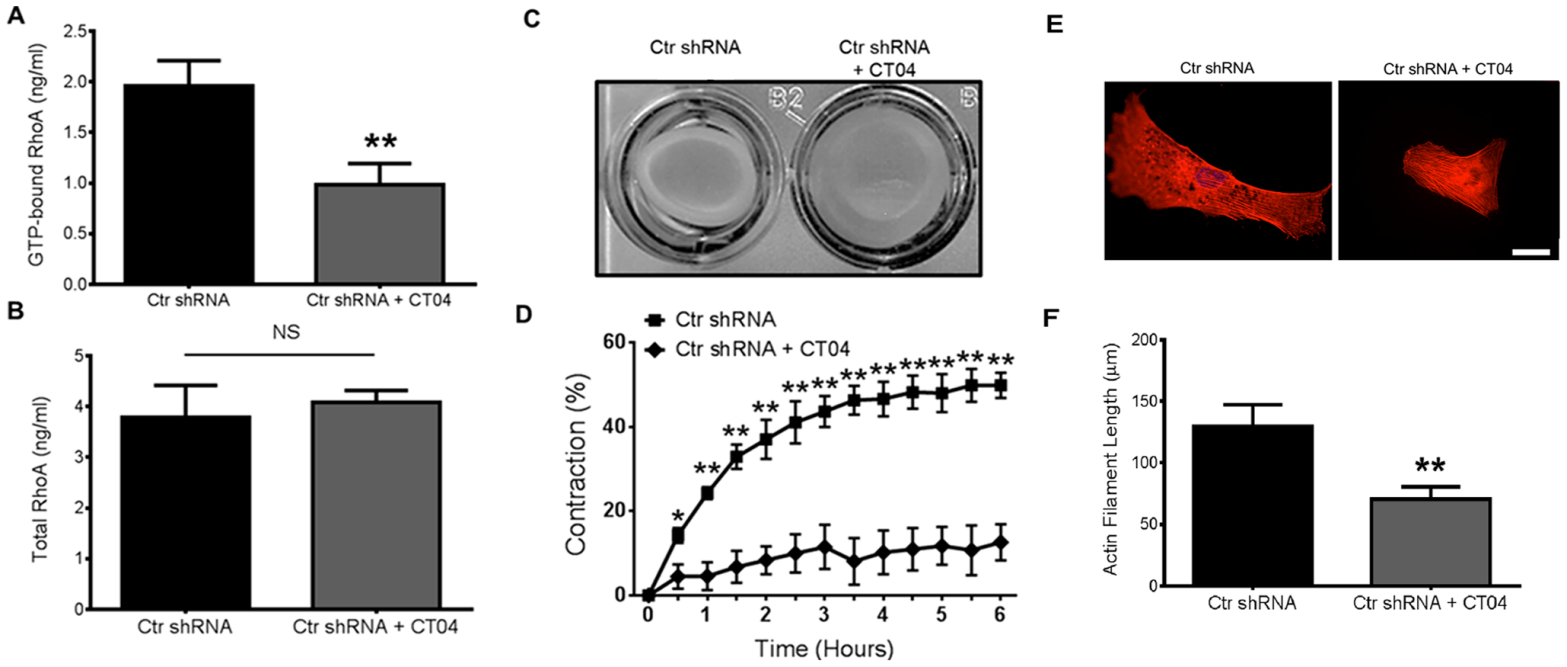
Suppression of active RhoA or knockdown of total RhoA in HAOSMC mimics the phenotype of CD9 shRNA HAOSMC. **A** and **B,** Quantification of the concentration of GTP-bound (active) and total RhoA in untreated or CT04 (2.0 µg/ml for two h) treated Ctr shRNA HAOSMC (n = 3; NS indicates no significant difference; **P<0.01 versus untreated cells). **C** and **D,** Representative image of collagen gel contraction after 6 h and quantification of collagen gel contraction from 0–6 h after treating Ctr shRNA HAOSMC with CT04 (2.0 µg/ml) for 2 h prior to releasing the sides of the solidified gel (n = 3/time point; *P<0.05, **P<0.01 versus CT04 treated cells at each time point). **E,** Representative immunofluorescently stained images of f-actin arrangement from three independently repeated experiments in untreated Ctr shRNA and Ctr shRNA treated with 2.0 µg/ml of CT04 for two h (scale bar, 50 µm). **F,** Actin filament length was quantified using ImageJ analysis software (n = 15 cells; **P<0.01).

To further attribute RhoA activity to HAOSMC capability to contract, we inhibited GTP-bound RhoA in Ctr shRNA HAOSMC using CT04. CT04 is a commercially available, cell permeable C3 transferase (C3T) derived from *C. botulinum* which works by inhibiting Rho GTPase isoforms by ADP-ribosylation but does not affect Rac1 of Cdc42 activity [19]. We confirmed that GTP-bound RhoA was significantly decreased after treatment with CT04 without affecting total RhoA levels (Fig. 5A and 5B). Contraction of CT04-treated Ctr shRNA cells was significantly reduced compared to the untreated cells (Fig. 5C). A significant difference in contraction was observed after 1 h and remained significant for the duration of the assay (Fig. 5D). Furthermore, treatment with CT04 significantly disrupted the elongated formation of stress fibers observed in the untreated cells (Figs. 5E and F).

## Discussion

This study aimed to further clarify the underexplored role of tetraspanin CD9 in regulating human VSMC physiology. We found that downregulating the expression of CD9 resulted in a pronounced morphologic and physiologic changes. The majority of cells lacking CD9 were significantly decreased in size and more rounded in shape. However, we did not observe any significant differences in the expression of the most commonly reported smooth muscle cell markers excepting for calponin, which was upregulated upon knocking down CD9. Previous reports generally confirm a switch to the dedifferentiated phenotype as indicated by a comprehensive decrease in the expression of two or more smooth muscle cell markers [20]–[22]. Our morphological data implied that the smooth muscle cells were in the dedifferentiated state, but the absence of a change in SM α-actin and SM22α and the increase in calponin mRNA expression impeded our effort to define the phenotype using traditional smooth muscle cell marker expression methods. Nevertheless, using smooth muscle cell markers to define phenotype is limited and often relies on the treatment of VSMC in low serum or serum free conditions or with the addition of exogenous TGFβ1 [23], [24]. It is an interesting observation that the simple downregulation of CD9 is sufficient to significantly induce the expression of calponin in cell cultured under identical conditions. Calponin was previously identified to be critical in vascular development a may be indirectly correlated to CD9 expression in VSMCs.

We explored the hypothesis that the observed morphologic differences may be due to a change in the cell cytoarchitecture due to the absence of CD9. Our observations suggest that knocking down CD9 may disrupt the coordinated signaling between the ECM and the cells that elicits actin arrangement. This suggestion is supported by the fact that while F-actin appears to be intact and at a similar density between the two cells types, the F-actin in CD9 shRNA cells lacks the parallel arrangement seen with the Ctr shRNA cells. We suspect that the circular arrangement of F-actin in CD9 shRNA HAOSMC is due to the cell's inability to spatially organize signaling from the ECM considering the role CD9 has in cellular membrane organization [25], [26]. The observed loss of contractility was consistent with a previous report demonstrating a direct correlation between RhoA expression and VSMC contraction [27]. However, this is only the second report that demonstrates CD9 regulates endogenous collagen gel contraction [11] and is the first to attribute this CD9-related phenomenon to actin arrangement and RhoA activity.

The CD9 shRNA cell phenotype was reversed upon restoration of RhoA activity signifying that the loss of RhoA activity is cause for the inability for actin to properly arrange and the cells to contract. Interestingly, treatment of CD9 shRNA HAOSMC with CN03 resulted in significantly more GTP-bound RhoA compared to untreated Ctr shRNA cells. Despite the increased active RhoA levels in these cells, the plots of contraction over time between the two groups were superimposable. We believe that the amount of total RhoA in the Ctr and CD9 shRNA HAOSMCs accounts for the limits of cell contraction. This idea is substantiated by our later findings that loss of GTP-bound RhoA results in a drastic loss of cell contraction without affecting total RhoA levels (Fig. 5C and 5D).

Rho GTPase activation is multifaceted; however, the literature suggests that integrins [28], [29] and tetraspanins [30]–[32] may regulate RhoA activation by outside-in signaling from the ECM. The expression of tetraspanin CD151 was recently demonstrated to upregulate the activation of small GTPases including Ras, Rac, and Cdc42, but not RhoA in an adhesion-dependent manner [33]. The authors demonstrated that the expression of tetraspanins CD9, CD81, or CD82 was not capable of inducing a similar response for Ras, Rac, or Cdc42 activation, but did not measure RhoA expression or activation for these tetraspanins [33]. These experiments were conducted on laminin, a well-characterized substrate for α3β1 and α6β1 integrins. These integrins closely associate with CD151 and have been previously demonstrated to activate Rac without affecting Rho [34]. On the other hand, the fibronectin-binding integrin, α5β1, selectively activates Rho [34], [35] and its expression is upregulated after arterial injury [36]. Our laboratory has previously demonstrated that CD9 associates with α5β1 integrin in multiple cell lines including VSMCs [6], [37]. Perhaps the expression and association of CD9 and α5β1 in VSMC pathologies stabilizes the interaction between the integrin and ECM to augment RhoA activation, and therefore, cell contraction. While our observations of contraction were performed in collagen gels, the potential importance of this interaction is highlighted by the findings that suppression of Rho kinase (ROCK), a direct effector of active RhoA, led to decreased neointimal formation in two animal models [38], [39] and prevented the formation of spontaneous hypertension in rats [40]. Pathologic SMC have been demonstrated to secrete fibronectin in culture [41]. The presence of CD9 may augment the pathologic condition in response to this endogenous production of fibronectin.

Evidence that CD9 may complex directly with GPCRs exists [42]. The authors confirm but do not reveal the association of additional tetraspanin and GPCR complexes in a cell-type specific manner [42]. More recently, Carloni et. al. reported that CD9 specifically colocalized with RhoA and the small heterotrimeric G protein subunit Gα13 in the absence of CD81 [43]. Our preliminary findings do not indicated differences between Ctr and CD9 shRNA HAOSMC p115 RhoGEF protein expression (Herr et. al., unpublished observations), which is a direct target of the Gα13 subunit [44]. Nonetheless, p115 RhoGEF activity has yet to be determined. Our current report expounds upon the idea that CD9 may regulate RhoA directly and contributes to the premise that different tetraspanins may regulate different aspects of Rho GTPase signaling.

Our study adds to the understanding of how tetraspanin CD9 contributes to human VSMC phenotypic transformation. We have demonstrated for the first time that the absence of CD9 alone is sufficient to effect major changes in the VSMC complex phenotype by regulating the arrangement of the actin cytoskeleton and the endogenous contraction of HAOSMC through the activity of RhoA. Thus CD9, through signaling mediators, integrins, and the reciprocal coordination of the cell cytoskeleton, is added to the list of influential factors that provide segue to VSMC phenotypic modulation. How CD9-induced modulation of cell contractile capabilities are involved in the pathophysiology of vascular injury response and atherosclerotic lesion development via the two proposed mechanism above remains to be established and is the current focus of our laboratory.
